# Multi-level considerations for optimal implementation of long-acting injectable antiretroviral therapy to treat people living with HIV: perspectives of health care providers participating in phase 3 trials

**DOI:** 10.1186/s12913-021-06214-9

**Published:** 2021-03-20

**Authors:** Andrea Mantsios, Miranda Murray, Tahilin S. Karver, Wendy Davis, Noya Galai, Princy Kumar, Susan Swindells, U. Fritz Bredeek, Rafael Rubio García, Antonio Antela, Santiago Cenoz Gomis, Miguel Pascual Bernáldez, Maggie Czarnogorski, Krischan Hudson, Nicki Walters, Deanna Kerrigan

**Affiliations:** 1 Public Health Innovation & Action, New York, NY USA; 2 ViiV Global Health Outcomes, London, England UK; 3 Johns Hopkins University, Baltimore, MD USA; 4 George Washington University, Washington, DC USA; 5 Georgetown University, Washington, DC USA; 6 University of Nebraska Medical Center, Omaha, NE USA; 7 Metropolis Medical, San Francisco, CA USA; 8 Hospital 12 de Octubre de Madrid, Madrid, Spain; 9 Hospital Clinico Universitario de Santiago de Compostela, Santiago de Compostela, Spain; 10 ViiV Healthcare, Madrid, Spain; 11 GSK, Madrid, Spain; 12 ViiV Healthcare, Research Triangle Park, NC USA; 13 ViiV Healthcare, London, UK

**Keywords:** HIV, ART, Long-acting injectable, Implementation, Health care providers, Clinical settings

## Abstract

**Background:**

Long-acting injectable antiretroviral therapy (LA ART) has been shown to be non-inferior to daily oral ART, with high patient satisfaction and preference to oral standard of care in research to date, and has recently been approved for use in the United States and Europe. This study examined the perspectives of health care providers participating in LA ART clinical trials on potential barriers and solutions to LA ART roll-out into real world settings.

**Methods:**

This analysis draws on two data sources: (1) open-ended questions embedded in a structured online survey of 329 health care providers participating in the ATLAS-2 M trial across 13 countries; and (2) in-depth interviews with 14 providers participating in FLAIR/ ATLAS/ATLAS-2 M trials in the United States and Spain. Both assessments explored provider views and clinic dynamics related to the introduction of LA ART and were analyzed using thematic content analysis. The Consolidated Framework for Implementation Research (CFIR) was drawn on as the conceptual framework underpinning development of a model depicting study findings.

**Results:**

Barriers and proposed solutions to LA ART implementation were identified at the individual, clinic and health system levels. Provider perceptions of patient level barriers included challenges with adhering to frequent injection appointments and injection tolerability. Proposed solutions included patient education, having designated staff for clinic visit retention, and clinic flexibility with appointment scheduling. The main provider concern was identifying appropriate candidates for LA ART; proposed solutions focused on patient provider communication and decision making. Clinic level barriers included the need for additional skilled individuals to administer injections, shifts in workflow as demand increases and the logistics of cold-chain storage. Proposed solutions included staff hiring and training, strategic planning around workflow and logistics, and the possibility of offering injections in other settings, including the home. Health system level barriers included cost and approvals from national regulatory bodies. Potential solutions included governments subsidizing treatment, ensuring cost is competitive with oral ART, and offering co-pay assistance.

**Conclusions:**

Results suggest the importance of multi-level support systems to optimize patient-provider communication and treatment decision-making; clinic staffing, workflow, logistics protocols and infrastructure; and cost-related factors within a given health system.

**Supplementary Information:**

The online version contains supplementary material available at 10.1186/s12913-021-06214-9.

## Background

Antiretroviral therapy (ART) to treat human immunodeficiency virus (HIV) is highly effective when taken regularly. Daily oral ART can help people living with HIV (PLHIV) to achieve and maintain life-long viral suppression, but requires consistent adherence over a life time [1]. Several decades into the use of ART, there is substantial evidence of sub-optimal adherence to oral ART across settings and populations [2]. Barriers to daily oral ART adherence include those imposed by characteristics of pill regimens and their side effects, health care systems and provider communication issues [3–5]. Significant psychosocial and structural barriers include substance use and mental health [6–9], inequitable gender norms and roles [7, 10], and stigma and discrimination associated with HIV and marginalized populations disproportionately affected by the epidemic [11, 12]. A new delivery method, long-acting injectable (LA) ART, offers an alternative to oral regimens which may address some of these adherence challenges. Clinical trials of a two-drug intramuscular (IM) long-acting regimen of Cabotegravir (CAB) and Rilpivirine (RPV) [13–16] have demonstrated that LA ART is non-inferior to daily oral ART, showing rates of viral suppression, treatment failure, and drug resistance similar to daily oral ART [17–19].

LA ART requires periodic injections, monthly or every 2 months, eliminating the need for taking daily pills. This may provide a more convenient and, for some PLHIV, a more private option. This new treatment modality may present the possibility of improved adherence to therapy and thus has the potential to improve individual level outcomes including viral suppression, and as a result, population level transmission dynamics [20]. Prior research has documented acceptability of LA ART among PLHIV [21–24] and satisfaction with LA ART among clinical trial participants [25–28]. In a qualitative study we conducted with patients and health care providers participating in the LATTE-2 phase 2 trial [25], providers were generally supportive of LA ART as a treatment option. However, they expressed clinic readiness concerns including the requirement for patients to attend more frequent clinic visits and the need for sufficient trained health care staff to administer injections [25]. These practical questions and concerns underscored the need for further research exploring provider perceptions of the feasibility of implementing LA ART and how potential challenges might be addressed. Given that LA ART has recently been approved for use in settings such as the United States and Europe [29, 30], work to optimize implementation is timely and strategic.

We conducted formative research in the context of clinical efficacy trials to assess potential barriers to LA ART implementation and possible solutions. In this qualitative study, we examine perspectives of health care providers using LA ART in clinical trials to assess challenges and opportunities for future roll-out of LA ART with the ultimate goal of optimizing implementation of LA ART delivery in routine clinical care and, in turn, improving the health outcomes of PLHIV.

## Methods

### Study design

We employed a multi-methods approach [31] combining two sources of data collection and two methods of data analysis for this qualitative study using a cross-sectional, exploratory design. Our analysis draws on data from both open-ended questions embedded in a structured online survey conducted with 329 health care providers from 13 countries participating in the phase 3 ATLAS-2 M trial and 14 in-depth interviews (IDIs) with health care providers in the United States and Spain participating in the ATLAS, ATLAS-2 M and FLAIR phase 3 clinical trials of LA ART [14–16]. ATLAS and ATLAS-2 M enrolled treatment-experienced participants while FLAIR enrolled treatment-naïve participants whose virus had been suppressed using coformulated abacavir/ lamivudine/ dolutegravir for 20 weeks. All three trials required participants to take oral CAB + RPV for 4 weeks prior to LA ART to ensure tolerability. ATLAS and FLAIR trials randomized participants to continue on oral ART or switch to monthly injections. ATLAS-2 M randomized participants to receive injections monthly or every 2 months.

We developed a semi-structured, in-depth interview guide (provided as Additional File 1) for health care providers in the United States and Spain participating in the ATLAS, ATLAS-2 M and FLAIR trials based on prior formative research conducted with providers in the phase 2b LATTE-2 study. The guide contained a series of open-ended questions aimed at exploring provider, clinic and health system issues related to LA ART. Fourteen semi-structured IDIs were conducted with health care providers using the guide to elicit their views on and experiences with LA ART. For IDIs in the U.S., three urban clinical sites were included: Washington, D.C., Omaha, Nebraska and San Francisco, California. In Spain, eight sites participated in the study from six diverse geographic locations, including major urban research hospitals as well as smaller and more rural clinics. Two of the sites were in Madrid, two were in Barcelona, and one site each were in Santiago de Compostela, La Coruña, Valencia, and Palma de Mallorca.

In parallel, we developed a structured online survey (provided as Additional File 2) to assess the feasibility of LA ART across a geographically diverse and larger sample of health care providers participating in a clinical trial of LA ART. The survey development was primarily based on the key themes raised by providers in our prior formative work with health care providers participating in the LATTE-2 study. Additional insights were gleaned from the literature, advisory boards, and pilot-testing with investigators from the ATLAS-2 M study. The survey was administered to providers in the ATLAS-2 M trial to further understand their views and clinic and health system dynamics related to the introduction of LA ART, including barriers to implementation and opportunities for roll-out. The survey included three open-ended questions asking 1) what providers considered to be the most significant barriers to successful implementation of LA ART, 2) how those barriers could be overcome, and 3) what would be needed for their site to implement LA ART once it is available. The survey was first developed in English and then translated into 7 languages: French, German, Italian, Korean, Russian, Spanish, and Swedish.

### Study participants and sample composition

Characteristics of interview participants and survey respondents are shown in Table 1. Of the 14 providers who participated in the IDIs, 6 were from the U.S. and 8 were from Spain. All individuals were involved in the phase 3 trials, either as study investigators (physicians) (*n* = 10) or study staff (e.g. study coordinators, nurses or other project staff) (*n* = 4) and all had contact with patients receiving LA ART as part of these trials. Spain and the U.S. and the specific clinics within these two countries were chosen for the qualitative study based on consideration of factors such as geographic diversity, the large number of people enrolled in the trials in those settings, and established on-the-ground partnerships.

**Table 1:** Sample Characteristics

	Online survey participants (*n* = 258)		In-depth interview participants (*n* = 14)	
		n (%)		n (%)
Region	Europe	158 (61%)	Spain	6 (43%)
	North America	61 (24%)	U.S.	8 (57%)
	East Asia and the Pacific	16 (6%)		
	Africa	13 (5%)		
	Latin America	10 (4%)		
Clinical role	Physician	156 (60%)	Physician	10 (72%)
	Nurse/Study Nurse/PA	64 (25%)	Nurse	2 (14%)
	Research Staff	29 (11%)	Administrator	2 (14%)
	Pharmacist	9 (4%)		
Prior LA ART trial involvement	1–2 clinical trials	134 (52%)	ATLAS/ATLAS-2 M	8 (57%)
	3 or more clinical trials	124 (48%)	FLAIR	6 (43%)

The online survey was sent to a total of 449 clinical trial staff, 329 (73%) of whom initiated the survey. Of those, 258 (78%) submitted responses to any of open-ended questions regarding implementation barriers and solutions. Providers participating in the survey who responded to the open-ended questions came from five geographic regions including: 61% from Europe (France, Italy, Germany, Russia, Spain, Sweden), 24% from North America (Canada, the United States), 6% from Asia and the Pacific (Australia, South Korea), 5% from Africa (South Africa), and 4% from Latin America (Argentina, Mexico). More than half of providers, or 60%, were physicians, 25% were nurses or physician assistants, 11% were research staff and 4% were pharmacists. Most of these providers had participated in more than one trial related to LA ART, with 52% having participated in one or two trials and 48% participating in three or more LA ART trials.

### Data collection and management

For IDIs, providers were identified by study investigators from the local trial sites. Written informed consent was obtained from all providers prior to the interviews which were conducted at the participating clinics in the U.S. and Spain. Interviews were conducted in the local language (English or Spanish) and lasted approximately 60 min. Interviews were audiotaped and transcribed verbatim in the language in which they were conducted. The study team used unique identifiers to label forms and audiotapes; no identifiers were included in transcriptions of the interviews. Transcripts were imported into Atlas.ti**©** [32] qualitative data analysis software for analysis. Interviews were completed between April and September 2018.

For the survey, study investigators received an email from the trial sponsor (ViiV Healthcare) describing the survey and requesting they identify eligible candidates from their sites. Potential participants were sent an email with information about the survey and the link to the online survey. Participants provided consent to participate on the first screen of the survey which was administered using Qualtrics© [33] software before any questions appeared. Providers were given 2 weeks to complete the survey and for those who did not respond, they were sent a reminder email. The survey took approximately 20 min for providers to complete and was conducted between February and May 2019.

### Analysis

#### In-depth interviews

IDI transcripts were analyzed using an iterative thematic content analysis approach [34]. A codebook was developed in English and used to inform the coding structure from a priori codes based on the original interview guide and study objectives. Additional domains emerging from the data were evaluated by two independent coders and the study investigators for relevance and accuracy. These were then added to the codebook during the process of refining the thematic coding structure. Transcripts were coded for both a priori and emergent domains of interest [35]. Throughout the coding process, saturation of themes was monitored, ensuring that thematic redundancy was established, and that little new information related to the primary domains was emerging [36]. It was expected there would be uniformity in clinician perspectives across sites in the two countries, and the homogeneous nature of the population would require a smaller sample [37]. With 10 of the 14 IDI participants serving as study investigators at their sites, most interview participants shared a specific role and thus had exposure to similar aspects of the administration of LA ART at their sites, resulting in a convergence of views in the data and saturation being achieved in the sample.

#### Survey responses

Textual data from open-ended survey responses were analyzed using a deductive content analysis approach. All data were reviewed for content and entered into a categorization matrix grouped by themes generated through the use of hierarchical codes [38]. The hierarchical coding frame was developed using the type of barrier and additional granularity for sub-codes when needed (e.g. injection frequency, scheduling). Validity of the matrix was assessed to confirm that categories adequately represented the concepts and the matrix captured what was intended [39]. Analytic summaries were written per country and per region to synthesize output. Responses were examined across countries and regions to assess similarities and differences and global themes were then extracted from across the summaries.

Comparisons were drawn between the two data sources to determine for which domains the data from the interviews and the survey converged. The level of convergence was high among the interview participants, even across the two country settings, and lower among survey participants both across and within countries (e.g. divergence in the data across providers from the same country as well as from one country to the next). These difference made it particularly compelling when we identified convergence between the survey data and the interview data and directed our analysis to focus on these key domains on which we found convergence between the two data sources. Themes extracted from IDIs were mapped onto the matrix of themes from survey responses and supporting quotes from IDIs were added. Based on thematic groupings from the code output and summaries of each data source, results were organized by patient, clinic, and systems level considerations. Given its emphasis on multi-level influences on implementation, we used the Consolidated Framework for Implementation Research (CFIR) [40] to help organize our findings. The CFIR provides a theoretical framing for using formative research to inform implementation of an innovation such as LA ART and includes the following five domains: individual, inner setting, outer setting, intervention and process. We drew on the CFIR to inform the development of a model situating findings within the established and well-understood domains of the individual, inner setting, and outer setting.

## Results

Across interview and survey data, health care providers identified potential barriers and possible solutions to implementing LA ART at multiple levels including: the individual level, thinking about their patients’ needs and preferences and their own role and attitudes as providers in this process; the clinic level, assessing clinic operations and structure; and the systems level, considering national regulations and health care coverage in their countries. Findings presented below include the key themes around which the data converged from both data sources and are organized by individual, clinic, and health system level.

For each level, we present a synopsis of the most frequent and salient responses from survey respondents and interview participants. Illustrative quotes from IDIs provide further nuance and description throughout the narrative and a table follows which summarizes potential barriers and proposed solutions from survey participants with direct quotes from responses to the open-ended survey questions.

### Individual level considerations

#### Provider perceptions of patient barriers and proposed solutions

At the individual level, providers perspectives on patient barriers focused on injection frequency and tolerability. Providers had concerns about the frequency of the injection appointments including patient non-adherence and the risk of resistance due to missed appointments, scheduling challenges, and clinic access, specifically distance to clinics and the challenge of patients not being able to return for a timely visit for each injection. Providers perceived injection tolerability to be another key barrier for patients, both in terms of pain at the time of the injection and injection site reactions following administration of treatment. An IDI participant in the U.S. highlighted concerns about both injection frequency and tolerability for patients saying:

*I think the biggest drawback is just, one, having to go to your doctor’s office every month and then, two, just the pain of an injection. It’s a lot more painful than swallowing a pill.* (U.S. study coordinator, IDI participant)

Capturing the concern around clinic accessibility, an interview participant in the U.S. discussed her experience in the trials with staff efforts needed to help patients adhere to appointments:

*I think the challenge is to be able to get them to be able to get to you. Some places are just not the easiest places to get to. I always think that my patients do well in clinical trials because of the contact with somebody that’s so constant. You don't show up, the study nurse calls you and says “Hey, where are you?”* (U.S. physician and PI, IDI participant)

Across interviews and survey responses, patient education came up as a proposed solution for both helping patients understand the importance of adhering to injection appointments and in tolerating injection-related pain. An in-depth interview participant in the U.S. described the need for patient education as follows:

*Have somebody able to educate [patients] as far as what to expect. Most of our patients who have gotten injectable medications, the first ones are pretty painful, but after that it keeps getting less and pretty soon they don't even really notice it anymore. I feel like having somebody who knows what information to tell them, kind of walking them through, telling them what to expect, during those first few injections, is important.* (U.S. nurse and study coordinator, IDI participant)

Another common proposed solutions to patient level barriers was to ensure clinic flexibility with appointment scheduling. One interview participant from the U.S. described the way her team has worked around scheduling challenges with trial participants at their site:

*A lot of times, we’ll start study visits earlier in the morning. Sometimes we'll come in at 7:00 or 7:30 to start seeing some of those patients that are working. We've had a couple of patients, “Hey, I'm going to Europe from this date to this date.” So we've had to do a little bit of creative work in communicating with the team about making it work with their injection schedules.* (U.S. nurse & study coordinator, IDI participant)

As shown in Table 2, additional solutions proposed by survey respondents included patient incentives, peer navigators, appropriate injection techniques to minimize pain, alternative locations for injection administration, and less frequent dosing (every 2 months versus monthly).

**Table 2:** Individual level barriers to LA ART implementation and proposed solutions

Themes	Provider Responses	
	Perceived Barriers	Salient Proposed Solutions with Representative Quotes
**Provider-perceived patient-level**		
**Adherence to frequent injection appointment**	“Attending 6-12 times/year instead of 2-3 and costs/inconvenience associated with this.” (Australian provider)	***Flexible scheduling, less frequent dosing*** “Longer opening hours, patient auto-injection, take it every 8 weeks.” (Swedish provider) “Having a 2 monthly injection would be better for patients to work into their timeline/lifestyle/work schedule” (Australian provider)
	“The patient needs to come more often compared to oral ART” (Swedish provider)	
	“Having to come for injection during regular opening hours (from 8h00 to 16h00 for example).” (Canadian provider)	***Increased access through multiple locations*** “Develop pharmacies with injectable ARV management capacity, similar to vaccinations.” (Argentinian provider) “Health centers close to patients' homes for medication administration.” (Spanish provider)
	“Having to comply with injection window” (Argentinian provider)	
	“The need to meet fixed administration deadlines” (Spanish provider)	***Patient education, support and guidance*** “Appropriate patient education on the treatment to ensure commitment to scheduled visits.” (French provider)
	“Getting patients with unstable lifestyles to come to clinic” (U.S. provider)	“Recall [smart phone] application for injection date.” (French provider)
	“Concerns over resistance in lost to follow up patients” (U.S. provider)	“Making sure clear communication with patient on what is required for medication.” (U.S. provider)
		***Information on bridging between LA and oral ART*** “Know better how to go from the oral route to the I.M. and vice versa in periods of different duration to better adapt ART to the needs of patients” (Spanish provider)
		“Flexibility to bridge with oral therapy when unable to come in for injection.” (U.S. provider)
**Tolerability of injections**	“Injection site pain, mostly related to RPV LA.” (Canadian provider)	***Train clinic staff on how to minimize reactions*** “Reporting in detail local side effects of development studies and facilitating administration guidelines to prevent them (if known).” (Spanish provider)
	“Possible local sequelae of the accumulation of IM injections for life.” (Argentinian provider)	***Advise patients on how to manage injection site reactions*** “Appropriate injection technique and patient education for pain management.” (U.S. provider)
	“Drug-drug interactions, injection-related side effects” (Italian provider)	
	“Fear of long-term effects at the puncture sites” (German provider)	***Patient-provider communication and patient support*** “Since I have had experience with patients afraid of the injections and have helped them successfully deal with this, I can share this with other patients who may want to go on the injectables but are afraid of the shots.” (U.S. provider)
	“Fear of injections” (Russian provider)	“Talking the patient through the whole process and what to expect”(Australian provider)
	“Needle shy patients, buttock injections” (U.S. provider)	“Offer a support platform for patients.” (Spanish provider)
**Provider-level**		
**Determining appropriate candidates**	“Selecting appropriate patients” (Australian provider)	***Patient-provider communication and decision-making*** “To ensure patient is very well informed & has been given plenty of time to consider this change.” (Australian provider)
	“High virus suppression rate for long-term candidates” (South Korean provider)	“Communication with the patient” (Russian provider)
	“Adequate selection of patient profile” (Spanish provider)	“Patient choice” (Argentinian provider)
	“Ensure proper choice of candidates” (Russian provider)	“Patient counselling & careful selection of appropriate patients.” (South African provider)
	U.S. “ Determination of which patient populations to prioritize, both in resource-rich and constrained settings, based on patient characteristics, adherence level, inadequate virologic suppression rates, cost constraints, and accurate cost projections” (U.S. provider)	“Carefully discuss options and benefits and challenges to determine suitability” (U.S. provider)
		“Respect patient preferences.” (Canadian provider)
	“Clinicians will likely be hesitant because of their concerns regarding the potential to do harm, which may make them less likely to want to offer the drug to their patients. Need to take the practitioner comfort level out of the equation.” (Canadian provider)	***Resources to support provider-patient communication and decision-making*** “Patient-doctor guide” (French provider)
		“Provider resources for coming off LA” (U.S. provider)
		“Well screener observant and available patients before proposing the injections and propose a provisional schedule of injections established in advance” (French provider)

#### Provider barriers and proposed solutions

The main challenge identified by providers was determining appropriate candidates for LA ART. Throughout the IDIs, providers reported mixed perspectives regarding whether “adherent” or “non-adherent” patients were the more appropriate LA ART candidates. For example, providers were concerned about patients who travel frequently and who may in turn not always be able to attend their injection appointments. This issue led them to consider placing such patients back on an oral regimen. Providers often expressed a tension between recognizing that non-adherent patients could be appropriate candidates, while balancing their understanding that patients under this treatment modality need to adhere to clinic appointments in order to receive their injections. This tension is captured in the following quote:

*The patient who is very badly adherent, this is the patient that I am afraid to use an injectable treatment on because that patient scares me if he/she is lost and appears three weeks later than he/she had to...The patient that would be theoretically a very good candidate [is] that patient who is a disaster in adherence. Because the injection is only one day, but if on that day he/she does not come and he/she disappears...* [Spain male physician, Sub-P.I., ATLAS/ATLAS-2M trials]

Nevertheless, there seemed to be a middle ground that could be found between these two seemingly mutually exclusive categories of “adherent” and “non-adherent” patients, from the perspective of providers who commented that it was about finding those patients who may have trouble taking pills; yet, are still able to come in for injection appointments on a set schedule. As shown in Table 2, proposed solutions among survey respondents focused on patient-provider communication and decision-making around appropriateness of LA ART including resources to assist with such conversations.

### Clinic level considerations: infrastructure and staffing

Clinic level barriers primarily related to the logistical challenges of increased volume of patients receiving LA ART injections. Providers’ key concerns were around capacity to meet demand including the need for additional staff and skilled individuals to administer the injections, shifts in workflow as demand increases, and the logistics of cold-chain storage. An interview participant in the U.S. described her concerns around how to manage logistics of providing LA ART to a large portion of the patient caseload at her site:

*We have 1200 people come to my clinic here, just for example, and if most of them had to come every month or even every other month and get an injection, I don’t know how we would physically be able to do that with the infrastructure that we have. Just space, the nurses, everything else. So just how to actually administer it. I think it’d be difficult to do in the context of a busy outpatient clinic.* (U.S. physician and PI, IDI participant)

Providers raised concerns around the time and effort that staff would need to dedicate to retention. An interview participant in the U.S. described the time and effort of one of her team members to get trial participants back to the clinic for their injection appointments:

*She really has to do a lot of retention work with keeping them coming to appointments; they don't show and you have to call them to keep coming back, they need that support.* (U.S. nurse and study coordinator, IDI participant)

Describing the need for adequate refrigeration and storage facilities for cold chain logistics, one U.S. interview participant said:

*I want to be able to have it stocked in my refrigerator where I keep my vaccines but in a way in which there's a long shelf life, I don't have to mess with it a lot. It has to come all preformed. Long shelf life and preformed.* (U.S. physician and PI, IDI participant)

A solution proposed by numerous survey respondents was to have a team of individuals to coordinate an injection program (e.g. designated staff for scheduling, injections, retention) with a dedicated clinic or injection rooms on site for administering LA ART. A few interview participants suggested utilizing nursing staff already administering intramuscular injections (e.g. for sexually transmitted infections (STIs)).

Many survey respondents across regions proposed offering injections in other settings such as pharmacies to address the potential for a surge in demand beyond what their clinic staff could handle. Several interview participants in the U.S. and Spain discussed the need to have LA ART administered in an array of clinical care settings such as community health centers, family practices, or dedicated HIV treatment centers. An interview participant in Spain described how the expansion of services to other clinical settings would aid with a potentially high influx of patients switching to LA ART:

*If, in the future, a very high number of patients go on to receive this treatment, surely it will have to be taken from the hospital and brought to the health centers because it will not be possible for us to have 900 patients coming here to get an injection. The nurse would not be sufficient….* [Spain physician and PI, IDI participant]

One U.S. interview participant who was very cognizant that reaching non-adherent patients was important, despite its difficulty, noted the potential similarities used for injectable antipsychotic medications that follow a home-based delivery model. This provider explained:

*So if you use the analogy from long-acting antipsychotics where these have been very successful, a lot of the models work well when they have a visiting nurse who will actually go to patients’ homes and give them the medicine and keep tabs on them and really help them with support in other areas, and those are the models that work well, and so we need to develop something like that for the patients that really can’t or won’t take medicine every day.* [U.S. physician and PI, IDI participant]

As seen in Table 3, other solutions proposed by survey respondents included staff training on workflow, logistics, injection procedures, and proper temperature conditions for LA ART. Additional solutions included providing incentives for staff involved in the administration of injections and establishing appropriate workflows and protocols.

**Table 3:** Clinic level implementation barriers to LA ART and proposed solutions

Themes	Provider Responses	
	Perceived Barriers	Salient Proposed Solutions with Representative Quotes
**Capacity to meet demand**	“Keeping track of timing/scheduling of patient appointments, window for injections & follow up with patients that miss injections.” (U.S. provider)	***Train clinic staff*** “Logistics collaboration, training of health personnel, access and infrastructure” (Argentinian provider)
	“Infrastructure in the centers for its application and conservation of the drug.” (Argentinian provider)	***Designate staff/space for injections*** “Dedicated clinic or team” (Italian provider) “Injection rooms and specialists dedicated to the provision of injections, providing incentives to the staff involved in the administration of injections” (South Korean provider) “We would need to organize a team to look at assigning personnel to be in charge of supply, education, administration, and patient retention.” (U.S. provider)
	“Staff coordination for delivery of injections.” (South Korean provider)	
	“Need of personnel and physical space for its administration” (Spanish provider)	
	“Clinic time demands for injections” (U.S. provider)	***Establish workflows and protocols*** “Suggested workflow example of a typical patient injection visit” (Australian provider) “Work the injection appointments into clinic flow/create template for scheduling injection visits; staff training (drug ordering and storage, administration of injections, follow up/window allowed for injections); pharmacy tech trained on prior authorizations/insurance” (U.S. provider)
	“The need for specialized administration personnel.” (Spanish provider)	
**Cold chain logistics**	“The need for a strict cold chain.” (Russian provider)	***Train clinic staff*** “Training in clinics to educate staff about temperature needed to keep medications at and importance of proper dosing and temperature of medications.” (U.S. provider)
	“Cold chain in rural areas” (South African provider)	
	“Cold chain issues. Drug only free at one pharmacy in Melbourne and cold chain would therefore be broken.” (Australian provider)	***Adequate refrigeration and storage facilities*** “Would potentially need medication sent by a courier if patients don't wish to pay for it to ensure cold chain maintained.” (Australian provider)

#### Health system level considerations: cost and regulatory approvals

Health system level barriers included issues around cost and the need for approvals from regulatory bodies. Survey respondents across all regions expressed concern around the cost of the drug, what portion of cost insurance would cover, and how reimbursement would work (Table 4). Respondents also focused on obtaining approvals from the appropriate authorities within their countries for dispensing the medication from hospital pharmacies and having LA ART added to nationally covered medications lists.

**Table 4:** System level implementation barriers to LA ART and proposed solutions

Themes	Provider responses	
	Perceived barriers	Salient proposed solutions with representative quotes
**Cost - equivalency to oral ART**	“Possible high costs.” (Argentinian provider) “Cost constraints, and accurate cost projections.” (U.S. provider) “Cost of drug to physicians for purchasing for the office” (U.S. provider)	***Competitive cost with oral ART*** “Pricing must be equivalent to standard therapy.” (German provider) “Provision of LA regimen free to the public sector” (South African provider) “Reduce the cost of drugs for countries with a generalized HIV epidemic. “(Russian provider)
**Cost - mechanisms of reimbursement**	“Reimbursement for the medication.” (German provider) “Potentially the overall cost for insurance carriers (i.e. doctor visits + injections + medication cost).” (U.S. provider) “Insurance issues, insurance reimbursement for clinic injection visits.” (U.S. provider)	***Government-subsidized treatment*** “Having adequate approvals with ADAP, Medicare, and commercial insurances.” (U.S. provider) ***Co-pay assistance*** “Copay cards or assistance for patients with and without insurance” (U.S. provider) ***Clear compensation guidelines for clinics*** “Clear guidelines regarding compensation for the increased number of visits that injection treatment generates, we would end up with our order and not receive reimbursement for our costs - in short, the economy controls and in this case the staff cost.” (Swedish provider)

Cost came up repeatedly throughout IDIs in Spain and the U.S. as well. While among U.S. interview participants, most providers appeared to assume that insurance companies would defray much of the costs associated with LA ART, the issue of co-pays was raised in both countries as a potential barrier, especially if LA ART was significantly more expensive than daily oral ART. Among Spain interview participants, given their universal government-run health care system, providers were unsure whether the government would support a wide-scale roll-out of LA ART. One Spain interview participant elaborated explaining that what “*worries [him] is that the cost is too high and the number of patients that can be included would be relatively low.”* (Spain, physician and PI, IDI participant). A U.S. interview participant described her concerns as follows:

*I have a great concern about that from the reimbursement perspective, because..there are certain things that I give here-- I actually take a loss on that, so to me that's another big area of worry, because I have to be practical about that…It has to…guarantee that I will not lose money. I don't need to make money giving it, but I cannot lose money giving it.* (U.S. physician and PI, IDI participant)

Proposed solutions from survey participants focused on affordable drug pricing, governments subsidizing treatment, co-pay assistance, a competitive market that could lead to reduction in drug prices, clear compensation guidelines, patient and clinic financial support and ensuring cost coordination between pharmaceutical companies and insurance carriers prior to roll-out. There were several country specific responses. For example, in the U.S., respondents proposed expanding Medicare to include LA ART and having adequate approvals with the AIDS Drug Assistance Program (ADAP) and commercial insurances while respondents from Mexico mentioned ensuring that LA ART enters the basic drug treatment list (“cuadro básico” in Spanish) allowed in the country.

Several of the previously mentioned solutions were proposed by providers when thinking about how to address cost barriers they anticipated their site would experience with LA ART. These included self-administration, alternative facilities for administration, and offering less frequent dosing. Proposed solutions for the barrier of getting approvals from regulatory bodies focused on simplifying the registration process for new drugs.

#### Multi-level model for optimal implementation of LA ART

**Fig. 1:**
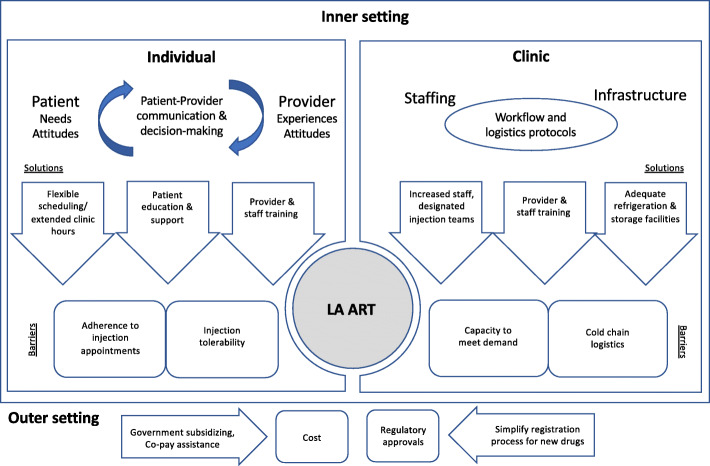
Multi-level Considerations for Implementing LA ART

To consolidate and organize our findings, we developed a multi-level model (Fig. 1) depicting the key considerations identified in this study to inform how providers, clinics and health systems may begin to consider and address potential challenges they may encounter when introducing LA ART into their clinical settings. We drew on the CFIR to inform the development of this model situating findings within the established and well-understood domains of the individual, inner setting, and outer setting [40]. At the center of the model is LA ART with its core components. Around it lie the individual and clinic levels with their borders curved to encircle LA ART illustrating the need for changes or adaptations to elements and processes at those levels to facilitate LA ART implementation.

Shown in rectangles surrounding the intervention are the potential barriers to implementation identified at each level by providers in this study. Their proposed solutions to address these barriers are shown in arrows pointing towards the intervention. Within the individual and clinic level, the key component critical to implementation as indicated by study findings is shown at top. These key components are patient-provider communication and decision-making at the individual level and workflow and logistics protocols addressing staffing and infrastructure challenges at the clinic level. Potential barriers and proposed solutions at the systems level are shown along the bottom of the figure separated from all factors in the inner setting indicating that they will be addressed by external entities separately from the more proximal implementation strategies that can be addressed through implementation plans.

## Discussion

Study findings reveal individual, clinic, and health system level considerations for administering LA ART as part of routine clinical care. This lays the groundwork for the development of needs assessments and implementation research across diverse types of clinical care facilities and geographic settings outside of an efficacy trial. Implementation science must play a key role in guiding the process of moving LA ART from clinical trials to real-world settings [41, 42]. Future studies can tailor implementation strategies to address some of the perceived barriers and proposed solutions identified in this study to optimize implementation effectiveness and success.

Findings underscore the need for a multi-level strategy to bring LA ART from trials to the field and have several implications for research and practice. At the system level, issues around cost and regulatory approvals rely on external bodies and will vary for each site depending on their national health system. Nonetheless, providers and clinic leadership will have to navigate how their sites are going to interact with and operate within established systems and mechanisms. At the individual and clinic level however, findings suggest several factors that should be considered in future implementation research and planning. Our model of Multi-level Considerations for Implementing LA ART (Fig. 1) highlights opportunities to make adaptations to elements and processes within the inner setting [40] at both the individual and clinic level which could address potential barriers identified in this study. For example, focusing on accommodating patient needs and preferences and providing supportive services and resources for patients may be important to implementation success, as may facilitating effective communication and shared decision-making between patients and providers regarding the appropriateness of an LA ART regimen. This work builds on our prior research documenting patient perspectives on LA ART [25–27] by adding provider prespectives on patient-centered approaches to improving uptake and adherence of this treatment modality. At the clinic level, assessing clinic readiness is paramount. The barriers and solutions identified in this study can be used to help guide clinic assessments of what resources will be needed to support the introduction of LA ART and to determine what is feasible at sites given their specific setting and circumstances.

Using the factors in our model of Multi-level Considerations for Implementing LA ART (Fig. 1), clinical sites can distill specific strategic implementation guidance on how to support patients and providers at the individual level and how to strengthen staffing and infrastructure at the clinic level. For example, at the individual level, patients and providers may benefit from using shared decision-making tools that guide their dialogues to help them reach a decision on whether LA ART is an appropriate fit. Providers may benefit from materials on and platforms for navigating clinical considerations of LA ART such as bridging with oral therapy, lead-in periods and pharmacokinetic tails. At the clinic level, checklists for refrigeration requirements and suggestions for effective and space-saving storage strategies would provide concrete support on logistical aspects. Training manuals including examples of typical patient injection visits and sample team work designation structures could help address workflow and staffing questions. Our model (Fig. 1) is also intended to offer insights and highlight priority areas for future implementation research. Future studies can further identify key strategic next steps by examining the factors highlighted here to inform effective implementation of LA ART in real-world contexts.

Based on the guidance providers expressed they would need to scale up LA ART, potential resources to support clinical sites in navigating this new treatment modality could include: training manuals on administering injections, medication management protocols, guidance to facilitate patient-provider communication and decision-making around appropriate candidates for LA ART. However, research on the sources of information that clinicians use has found that colleagues often rank as primary information sources and that informal learning is widespread [43–46], suggesting the importance of internal champions at individual sites. Individuals who can actively promote the LA ART implementation process [40] and facilitate knowledge sharing among staff and clinicians could potentially help sites adopt this new treatment modality.

As indicated by several survey participants (Table 2) (e.g. “offer a support platform for patients”, “recall [smart phone] application for injection date”, “online peer navigators”), the use of technology and online resources should be explored as an opportunity to provide what was perceived as much needed patient support for LA ART delivery. Evidence of such strategies has shown to be successful in encouraging appointment adherence [47, 48], fostering virtual health communities among chronic-disease patients [49], and providing support networks for stigmatized populations [50]. With the potential to address some of the key barriers providers perceived to patient success on LA ART, these strategies warrant further consideration and research.

Reflecting on the findings from this study, there are several broader implications to consider. Providers’ concerns about cost raise important questions around appropriate candidates given that, from a health system perspective, it may be necessary to potentially prioritize LA ART for those with the greatest unmet need. Thus, research on appropriate candidates for this treatment modality [51] is critical to help determine which patient populations to prioritize, both in resource-rich and constrained settings. Given recent results from HIV Prevention Trials Network (HPTN) 083 demonstrating that long-acting injectable CAB as LA pre-exposure prophylaxis (PrEP) is highly effective [52], findings from this study may also highlight important considerations for delivery of LA PrEP outside of clinical trials. Finally, given the current climate in which COVID-19 has significantly impacted many health systems around the world, launching a novel treatment such as LA ART brings additional challenges. In addition to the current pandemic exacerbating challenges identified in this study as clinic personnel are stretched and tasks are multiplied, individuals’ healthcare priorities are shifting as they assess personal risk and safety in accessing care at clinics. These considerations should be taken into account for thoughtful roll-out of this treatment modality in the current context.

This study had several limitations. First, the possibility for bias exists in both samples of data used in this analysis as all providers were participating in a clinical trial and thus their views and experiences may reflect a potential openness and predisposition to the acceptability and use of LA ART. Second, the sample is not representative as participants were disproportionately from the West (U.S. and Europe) and certain regions were represented by only one or two countries. Given that the perspectives of low- and middle-income countries (LMICs) were underrepresented in this study, considerations for effective LA ART in these settings will need to be further explored and cognizant of local contexts, since health system challenges and opportunities may be different. Additionally, sites participating in clinical trials are likely more prepared with the needed resources to begin LA ART implementation than more rural sites or even urban sites in resource-constrained settings. Finally, the current study is formative research not conducted in a real-world sample; rigorous implementation research should be carried out to examine direct translation to routine care. In an effort to avoid common pitfalls of semi-structured interviews including the possibility of bias introduced by the interviewer, we relied on experienced qualitative researchers trained in using a flexible guide and with working knowledge of the topic so as to establish trust, rapport, and active listening on the part of the interviewer with the participant. The researchers utilized memo writing to document thoughts, comparisons, and questions, and to reflect on interviewer perceptions to manage biases, assumption, and reactions to the data. This study had several strengths including that findings were validated through triangulation as we utilized data from different sources collected from participants in different regions of the world. Additionally, we found high levels of convergence across the two data sources on the key domains reported here.

## Conclusion

Study findings highlight the importance of a multi-level strategy to bring LA ART from clincal trials to routine care. Results suggest the importance of multi-tiered support systems to optimize patient-provider communication and treatment decision-making; clinic staffing, workflow, logistics protocols and infrastructure; as well as access and cost-related factors within a given health system. Key considerations identified in this study can inform future implementation research and plans for scale-up.

## Supplementary Information

**Additional file 1.**

**Additional file 2.**

## Data Availability

The datasets used and/or analysed during the current study are available from the corresponding author on reasonable request.

## Abbreviations

ADAP: AIDS Drug Assistance Program
ART: Antiretroviral therapy
ATLAS: Antiretroviral Therapy as Long Acting Suppression Trial
ATLAS-2 M: Antiretroviral Therapy as Long Acting Suppression every 2 Months Trial
CAB: Cabotegravir
CFIR: Consolidated Framework for Implementation Research
COVID-19: Coronavirus disease 2019
FLAIR: First Long-Acting Injectable Regimen Trial
HIV: Human immunodeficiency virus
HPTN: HIV Prevention Trials Network
IDI: In-depth interviews
IM: Intramuscular
LA ART: Long-acting injectable antiretroviral therapy
LATTE-2: Long-Acting Antiretroviral Treatment Enabling Trial 2
PI: Principal Investigator
PLHIV: People living with HIV
PrEP: Pre-exposure prophylaxis
RPV: Rilpivirine
STI: Sexually transmitted infection
US: United States
