# Supplementary material for: Multi-level considerations for optimal implementation of long-acting injectable antiretroviral therapy to treat people living with HIV: perspectives of health care providers participating in phase 3 trials
Source: BMC Health Serv Res. 2021 Mar 20;21:255. doi: 10.1186/s12913-021-06214-9 (PMC7980753; doi:10.1186/s12913-021-06214-9)
Supplement: Supplementary file 1 — Additional file 1. [file 12913_2021_6214_MOESM1_ESM.docx]

**Interviewer**: Collect demographic data on the
Participant Information Sheet before beginning interview.

*Thank you for taking the time to speak with me today. We are interested in hearing your perspectives and experiences as a clinical provider of patients on long-acting injectable (LA) ART. I’d like to start by talking a bit about how you first became aware of the LA option…*

**Initial awareness of LA ART**

- How did you first become aware of LA ART as an HIV treatment option?
  - Probe: When did you first become aware?
  - Probe: Who shared this information with you?
  - Probe: Where is this person based/located?
- From where else did you receive information regarding LA ART?
  - Probe: Conferences, professional groups, internet, etc.
- Tell me about your initial thoughts about LA ART?
  - Probe: What concerns did you have?
  - Probe: What other considerations went through your mind in terms of whether LA ART might make sense for your patients?
  - Probe: What did you see as the benefits of LA ART?
  - Probe: What did you see as the barriers?
- What and who would you say have been the biggest influencers on your thinking and decision to prescribe LA ART to your patients?
  - Probe: KOLs, local advocates, evidence based literature

**Decision to switch to LA ART**

- How many patients do you currently have on LA ART?
- How did those patients come to be on LA ART?
- Tell me about the initial conversations you had with those patients?
  - Probe: Who initiated those conversations?
  - Probe: What did you discuss?
  - Probe: What were the main questions/concerns you heard from those patients about switching from oral to injectable treatment?
- What made you feel like they were good candidates for LA ART?
- What would you say were the most important reasons for switching the patient to LA ART from an oral regimen?
  - Probe: Did the most important reasons vary between you and the patient? If yes, how so?

**Candidates and criteria for LA ART**

- What would be an appropriate patient to switch to LA ART?
  - Probe: Are there specific drug regimens where LA ART is more appropriate?
- What other patient profile or specific demographic or clinical characteristics need to be considered in a potential switch to LA ART?
  - Probe: gender, age, other behaviours (substance use, etc.)
- What are your biggest concerns or worries about LA ART?
  - Probe: Any concerns about effectiveness of LA ART vs. non-injectable therapy?
  - Probe: How would you say that LA ART performance compares?
- What would make you stop or pull a patient off LA ART?
  - Probe: Beyond side effects, what else?
- How do you plan on introducing LA ART to your patients in the future?
  - Probe: What do you think could help other people living with HIV make a smooth transition to LA ART?

**Experience switching patients to LA ART**

- Tell me about the first patient you had on LA ART…
  - Probe: Describe the decision-making process for me…
  - Probe: Did you speak to anyone else e.g. other clinicians, KOLs you know who have made this switch before
- Tell me about the most recent patient you placed on LA ART…
  - Probe: Describe the decision-making process for me…how was that experience different from the first patient you placed on this regimen?
- What types of changes have you observed that patients experience with the switch?
  - Probe: Initial reactions from your patients?
  - Probe: What are the most common side effects?
  - Probe: What about later on, do the side effects increase or decrease?
- What types of concerns have patients had?
  - Probe: How did you address those concerns?
- Tell me about any other negative experiences – treatment failure, patient discontinuation or compliance, etc.
  - Probe: How did you manage those issues?
- What types of benefits do you observe that patients experience?
  - Probe: Reduced side effects?
- What other types of benefits have you observed patients experience?
  - Probe: psychological, emotional benefits? lifestyle?
- What types of support and communication has been important for patients during the switch?
  - Probe: What kinds of questions have they brought to you?
  - Probe: What kinds of support have they expressed they needed?
- Overall, how would you describe your experience with LA ART?
- What else would you want to share about your experience switching patients to LA ART from oral dose regimens?

*Thank you for your time and insights. We really appreciate this important information.*
