# Supplementary material for: Multi-level considerations for optimal implementation of long-acting injectable antiretroviral therapy to treat people living with HIV: perspectives of health care providers participating in phase 3 trials
Source: BMC Health Serv Res. 2021 Mar 20;21:255. doi: 10.1186/s12913-021-06214-9 (PMC7980753; doi:10.1186/s12913-021-06214-9)
Supplement: Supplementary file 2 — Additional file 2. [file 12913_2021_6214_MOESM2_ESM.docx]

**Long-acting Injectable ART Implementation and Roll-out Survey**

**Survey Introduction**: As you know from your participation in clinical trials of long-acting cabotegravir + rilpivirine (CAB + RPV LA), ViiV Healthcare has been developing intramuscular injections of long-acting injectable anti-retroviral drugs.

**Goal**: Develop a better understanding from investigators, study coordinators, and other clinical care providers such as physicians and nurses involved in the trials of the benefits, barriers, and issues around the rollout of CAB + RPV LA.

**Purpose**: The information gathered in this survey will be used to inform the future implementation and roll-out of injectable therapies for HIV patients in real-world settings and routine care outside the realm of clinical trials.

**Consent to participate:** This is an anonymous survey. Participating in this survey is completely voluntary. The data collected through this survey will be analyzed and used in an aggregate fashion to describe trends in perspectives.

Please click “yes” if you agree to participate or “no” if you decline to participate.  Yes  No

Clinical site information:

Country: __________________ City: __________________ Name of clinic: __________________

Provider information:

My clinical role is:

 Physician

 Nurse

 Pharmacist

 Other medical personnel, please specify: _______________

I have been involved in the following CAB + RPV LA trials (check all that apply):

 LATTE

 LATTE2

 ÉCLAIR

 ATLAS

 FLAIR

 ATLAS 2M

1. **Logistics of Roll-out**

For the questions in this section, consider if CAB + RPV LA were to come onto the market outside of a research setting and was approved in your country. Please provide your thoughts on the practical issues around who, where, and how CAB + RPV LA would be implemented.

1. Overall, how feasible would it be to facilitate administering monthly injections of CAB + RPV LA at your clinic?

 Very feasible

 Somewhat feasible

 Not very feasible

 Not at all feasible

1. Please indicate how feasible it would be to administer injections of CAB + RPV LA to patients at your clinic every **4 weeks** considering the following:

|  | Very feasible | Somewhat feasible | Not very feasible | Not at all feasible | Don’t know |
| --- | --- | --- | --- | --- | --- |
| Space for administering injections |  |  |  |  |  |
| Personnel to administer injections |  |  |  |  |  |
| Clinic flow to accommodate administration of injections |  |  |  |  |  |
| Cold chain logistics |  |  |  |  |  |
| Refrigeration of the drug on site |  |  |  |  |  |
| Space for stocking and storing the drug |  |  |  |  |  |
| Maintaining stock given evolving number of patients requiring injections at any given point |  |  |  |  |  |
| Logistics of following up with patients for appointments |  |  |  |  |  |
| Other, please specify: |  | | | |  |

1. Now please indicate how feasible it would be to administer injections of CAB + RPV LA to patients at your clinic every **8 weeks** considering the following:

|  | Very feasible | Somewhat feasible | Not very feasible | Not at all feasible | Don’t know |
| --- | --- | --- | --- | --- | --- |
| Space for administering injections |  |  |  |  |  |
| Personnel to administer injections |  |  |  |  |  |
| Clinic flow to accommodate administration of injections |  |  |  |  |  |
| Cold chain logistics |  |  |  |  |  |
| Refrigeration of the drug on site |  |  |  |  |  |
| Space for stocking and storing the drug |  |  |  |  |  |
| Maintaining stock given evolving number of patients requiring injections at any given point |  |  |  |  |  |
| Logistics of following up with patients for appointments |  |  |  |  |  |
| Other, please specify: |  | | | |  |

1. In general, which settings would be most appropriate for administering CAB + RPV LA?

|  | Very appropriate | Somewhat Appropriate | Neither Appropriate nor Inappropriate | Not appropriate | Not Applicable |
| --- | --- | --- | --- | --- | --- |
| HIV-specialty clinic |  |  |  |  |  |
| Primary care clinic |  |  |  |  |  |
| Community health center |  |  |  |  |  |
| Hospital outpatient |  |  |  |  |  |
| Retail pharmacy healthcare clinic |  |  |  |  |  |
| Home health care |  |  |  |  |  |
| Other, please specify: |  | | | |  |

1. In general, which individuals are best suited for administering injections of CAB + RPV LA? (Check those that apply)

|  | Physicians |
| --- | --- |
|  | Nurses |
|  | Pharmacists |
|  | Other medical personnel, please specify: _________ |
|  | Other individuals, please specify: _________ |

1. What were some of the logistical barriers your patients faced in adhering to the clinic appointments during the trial? (check all that apply)

    Travel burden (e.g. distance to clinic)
    Time burden (e.g. waiting time, duration of appointment)
    Cost burden (e.g. public transport, parking fees)
    Burdened by number of appointments (e.g. separate appointments for injection and clinical visit)
    Travel for work or holiday  Instability (moving around a lot or no home)  Moved out of the area
    Other (specify: __________)
2. **Clinical management**For the questions in this section, please provide your perspective on some of the issues around the clinical management of CAB + RPV LA and how it compares to an oral regimen.
3. If you were considering initiating a long-acting regimen for a patient, how concerned would you be about the following challenges?

|  | Very concerned | Somewhat concerned | Not very concerned | Not at all concerned |
| --- | --- | --- | --- | --- |
| The oral lead-in phase before starting injections |  |  |  |  |
| Risk of resistance for patients not adherent to injections |  |  |  |  |
| Patients not returning to clinic for injection appointments |  |  |  |  |
| Taking a patient off CAB + RPV LA and switching to oral ART |  |  |  |  |
| Drug interactions and comorbidities (e.g. TB, HCV) |  |  |  |  |
| Patients moving out of the area |  |  |  |  |
| Patients switching to a different provider |  |  |  |  |
| Other (specify: __________) |  |  |  |  |

1. From your perspective, how necessary is it to have the oral lead-in phase before transitioning a patient to CAB + RPV LA?

 Absolutely necessary (Skip to Q10)
 Helpful but not necessary (Skip to Q10)
 Not at all necessary
 No opinion (Skip to Q10)

1. Why do you think the oral lead-in phase is not necessary?

____________________________________________________________________

1. How serious of an issue were the injection site reaction for you as a provider during the trial?

 Very serious
 Somewhat serious
 Not very serious

 Not at all serious

1. How serious of an issue do you think the injection site reaction were from the patients’ perspective during the trial?

 Very serious
 Somewhat serious
 Not very serious

 Not at all serious

1. Do you administer injections as part of your role in the trials?

 Yes
 No (Skip to Q16)

1. How many injections have you administered in the course of the ViiV trials you’ve been involved in (e.g. ÉCLAIR, LATTE, LATTE2, ATLAS, FLAIR, ATLAS 2M)?
   1. ≤ 20
   2. 21-50
   3. > 50
   4. I do not administer injections (skip to Q15)
2. What techniques are most useful to help minimize injection pain for patients (check all that apply?)?
   1. Medication at room temp
   2. Hot/cold packs
   3. Pain relievers
   4. Relaxing muscle
   5. Use topical lidocaine to numb the site prior to injection
   6. Other, please specify: _____________
3. What do you tell patients to do if they have an injection site reaction or any other post-injection pain? (select all that apply).

|  | Never | Sometimes | Often | Always |
| --- | --- | --- | --- | --- |
| Take over-the-counter pain-reliever |  |  |  |  |
| Come in to the clinic to be examined |  |  |  |  |
| Call the clinic to have questions answered by phone |  |  |  |  |
| Utilize virtual medicine options to communicate (e.g. online chat, on-screen consultation) |  |  |  |  |
| Other (specify: __________) |  |  |  |  |

1. For each of the following issues related to the clinical management of HIV patients on ART, please indicate if you would be more concerned if managing CAB + RPV LA compared to oral ART.

|  | More concerned with CAB + RPV LA | More concerned with oral ART | No difference  in concern |  |  |
| --- | --- | --- | --- | --- | --- |
| Adherence |  |  |  |  |  |
| Resistance |  |  |  |  |  |
| Drug interactions |  |  |  |  |  |
| Switching regimens |  |  |  |  |  |

1. While working on the CAB + RPV LA clinical trials, to what extent have your concerns changed on the 6 dimensions below?

|  | Less concerned now than I was before | More concerned now than I was before | No change in my views |  |
| --- | --- | --- | --- | --- |
| Patients adhering to injection appointments |  |  |  |  |
| Risk of resistance if patient not adherent to injections |  |  |  |  |
| Oral lead-in phase before injections |  |  |  |  |
| Side effects of the drug |  |  |  |  |
| Drug-drug interactions |  |  |  |  |
| Switching regimens (e.g. to CAB + RPV LA from oral or vice versa) |  |  |  |  |

1. Have you had experience transitioning a patient from CAB + RPV LA to a daily oral ART?

 Yes
 No (skip to Q20)

1. Thinking about transitioning a patient from CAB + RPV LA to a daily oral ART, how concerned are you about the following aspects?

|  | Not at all concerned | Not very concerned | Somewhat concern | Very concerned |
| --- | --- | --- | --- | --- |
| Timing of switching from CAB + RPV LA to daily oral ART |  |  |  |  |
| Understanding the combinations of medications to switch to |  |  |  |  |
| Reconciling patient desire to switch to oral when CAB + RPV LA may be addressing patient’s adherence barriers |  |  |  |  |
| Other (specify: __________) |  |  |  |  |

1. **Patient interest and provider readiness**

In this section, please consider whether patients not involved in CAB + RPV LA clinical trials are aware of an injectable ART regimen. Then consider your own preparedness to begin prescribing CAB + RPV LA outside of a clinical trial setting.

1. What proportion of your patients do you believe are appropriate candidates for CAB + RPV LA?

 None (0%)
 Very few (1-10%)
 Few (11%-25%)
 Some (25%- 50%)
 Many (>50%)

1. What proportion of your patients (not in clinical trials) have **asked about** the injectable ART option?

 None (0%)
 Very few (1-10%)
 Few (11%-25%)
 Some (25%- 50%)
 Many (>50%)

1. What type of information are your patients (not in clinical trials) asking about regarding to CAB + RPV LA (check all that apply)?

 Procedural (e.g. how often, how administered)
 Clinical (e.g. how it works in the body, possible side effects)
 Comparison to oral ART (e.g. pros and cons of each)

 Availability (e.g. is it available, how can someone get access to it)

 Cost/co-pay

 Research (e.g. how effective, data from longitudinal studies)
 Other; specify _____________________

1. If CAB + RPV LA were to come onto the market outside of a research setting tomorrow, how ready are you to begin prescribing it to your patients?

 Very ready
 Somewhat ready
 Not very ready
 Not at all ready

1. What information/guidance would you like regarding the implementation of CAB + RPV LA in clinical settings? (check all that apply)

 Checklists for cold-chain issues and storage of the drug on site

 Sample clinic flow for implementing CAB + RPV LA injections at a clinical site

 Training materials for clinical staff administering CAB + RPV LA injections

 Written materials on CAB + RPV LA to give to patients

 A toll-free number to call for more information

 A website with information, patient and provider perspectives, live chat

 Assistance with determining insurance coverage and reimbursement (US only)

 Other, specify: _____________________

1. **Benefits, Risks and Appropriate Candidates**

In this section, please focus on the positive and negative aspects of CAB + RPV LA for your patients in the trial(s).

1. Overall, how important are the following benefits of an injectable ART regime for your patients?

|  | Very important | Somewhat important | Not very important | Not at all important |
| --- | --- | --- | --- | --- |
| Convenience |  |  |  |  |
| Reduced side effects related to oral ART |  |  |  |  |
| Reduced drug/food interactions |  |  |  |  |
| Easier management of concomitant diseases (e.g. TB, Hep B, HCV, diabetes) |  |  |  |  |
| Reduced stigma |  |  |  |  |
| Other psychological/emotional benefits (e.g. eliminating daily reminder of living with HIV) |  |  |  |  |
| Privacy/confidentiality |  |  |  |  |
| Lifestyle (e.g. no concern of pills seen in home, during travel) |  |  |  |  |
| Increased contact with health care provider |  |  |  |  |
| Other, please specify |  | | | |

1. How likely are the following issues to influence you selecting an injectable ART regimen?

|  | Very likely | Somewhat likely | Not very likely | Not at all likely |
| --- | --- | --- | --- | --- |
| Injection site reactions |  |  |  |  |
| Secondary effects |  |  |  |  |
| Risk of resistance if not adherent to injections |  |  |  |  |
| Drug interactions with comorbidities (e.g. TB treatment) |  |  |  |  |
| Interactions with non-prescription natural/herbal remedies |  |  |  |  |
| Interactions with methadone, narcan |  |  |  |  |
| Frequency of visits (adherence) |  |  |  |  |
| Cost/access to meds |  |  |  |  |
| Less discretion if have to go somewhere/leave work every month to get injection |  |  |  |  |
| Other, specify |  |  |  |  |

1. Who do you think are the most “appropriate” patients for an injectable regimen?

|  | Very appropriate | Somewhat appropriate | Not appropriate |
| --- | --- | --- | --- |
| Patients with personal preference for injections |  |  |  |
| Young people (ages 18-24) |  |  |  |
| Substance user |  |  |  |
| Unstable lifestyle (e.g. homeless, psychiatric comorbidity, disabled) |  |  |  |
| Active lifestyle (e.g. frequent travel) |  |  |  |
| Non-adherent to daily oral ART |  |  |  |
| History of side effects |  |  |  |
| Treatment fatigue/treatment holidays |  |  |  |
| Prior treatment failure on oral regimens |  |  |  |
| Prior resistance on oral regimens |  |  |  |
| Other, specify |  |  |  |

Finally, please share your overall thoughts on the key opportunities and barriers to implementation of CAB + RPV LA.

1. In your perspective, what are the 3 most significant opportunities CAB + RPV LA presents?

___________________________________________________________________________________________

1. In your perspective, what are the 3 most significant barriers to implementation of CAB + RPV LA?

___________________________________________________________________________________________

1. In your perspective, how can we best overcome the barriers listed in your previous response?

___________________________________________________________________________________________

1. What would need to happen at your site for CAB + RPV LA to be implemented tomorrow?

____________________________________________________________________

____________________________________________________________________

____________________________________________________________________

*Thank you for taking the time to complete the survey.
This information will be used to help provide quality treatment and care for individuals living with HIV.*
